# A Cotton Lignin Biosynthesis Gene, *GhLAC4*, Fine-Tuned by ghr-miR397 Modulates Plant Resistance Against *Verticillium dahliae*

**DOI:** 10.3389/fpls.2021.743795

**Published:** 2021-11-18

**Authors:** Taiping Wei, Ye Tang, Pei Jia, Yanming Zeng, Bingting Wang, Pan Wu, Yonggang Quan, Aimin Chen, Yucheng Li, Jiahe Wu

**Affiliations:** ^1^School of Resources and Environmental Engineering, Anhui University, Hefei, China; ^2^State Key Laboratory of Plant Genomic, Institute of Microbiology, Chinese Academy of Sciences, Beijing, China; ^3^The Key Laboratory for the Creation of Cotton Varieties in the Northwest, Ministry of Agriculture, Join Hope Seeds Co. Ltd., Changji, China

**Keywords:** miR397, *GhLAC4*, lignin, *Gossypium hirsutum*, *Verticillium dahliae*

## Abstract

Plant lignin is a component of the cell wall, and plays important roles in the transport potential of water and mineral nutrition and plant defence against biotic stresses. Therefore, it is necessary to identify lignin biosynthesis-related genes and dissect their functions and underlying mechanisms. Here, we characterised a cotton LAC, *GhLAC4*, which participates in lignin biosynthesis and plant resistance against *Verticillium dahliae*. According to degradome sequencing and GUS reporter analysis, ghr-miR397 was identified to directedly cleave the *GhLAC4* transcript through base complementary. *GhLAC4* knockdown and ghr-miR397 overexpression significantly reduced basal lignin content compared to the control, whereas ghr-miR397 silencing significantly increased basal lignin levels. Based on staining patterns and GC/MS analysis, *GhLAC4* acted in G-lignin biosynthesis. Under *V. dahliae* infection, we found that G-lignin content in ghr-miR397-knockdowned plants significantly increased, compared to these plants under the mock treatment, while G-lignin contents in *GhLAC4*-silenced plants and ghr-miR397-overexpressed plants treated with pathogen were comparable with these plants treated with mock, indicating that *GhLAC4* participates in defence-induced G-lignin biosynthesis in the cell wall. Knockdown of ghr-miR397 in plants inoculated with *V. dahliae* promoted lignin accumulation and increased plant resistance. The overexpression of ghr-miR397 and knockdown of *GhLAC4* reduced lignin content and showed higher susceptibility of plants to the fungal infection compared to the control. The extract-free stems of ghr-miR397-knockdowned plants lost significantly less weight when treated with commercial cellulase and *V. dahliae* secretion compared to the control, while the stems of ghr-miR397-overexpressed and *GhLAC4*-silenced plants showed significantly higher loss of weight. These results suggest that lignin protects plant cell walls from degradation mediated by cellulase or fungal secretions. In summary, the ghr-miR397-*GhLAC4* module regulates both basal lignin and defence-induced lignin biosynthesis and increases plant resistance against infection by *V. dahliae*.

## Introduction

Angiosperm lignin, a type of polymer deposited in plant cell walls, plays a role in plant mechanical support, mineral and water transport, and resistance to pests and pathogens (Abdel-Ghany and Pilon, 2008; Santiago et al., 2013; Wang et al., 2014; Mottiar et al., 2016). Lignin is a complex phenolic polymer that mainly contains guaiacyl (G) units, syringyl (S) units, and trace ρ-hydroxyphenyl (H) units, which are polymerized from monolignol precursors, namely ρ-coumaryl alcohol, coniferyl alcohol, and sinapyl alcohol (Berthet et al., 2011; Zhao et al., 2013). Monolignol synthesis belongs to a branch of the phenylpropanoid pathway that occurs in the cytosol. Monolignols are transported from the cytosol to the apoplast, where they are oxidised to generate lignin by laccase (Vanholme et al., 2008).

Plant laccases (LAC, EC. 1.10.3.2), which belong to the blue copper oxidase/ρ-diphenol:dioxygen oxidoreductase family, are widely distributed in higher plants, including gymnosperms, monocots, and eudicots (McCaig et al., 2005; Turlapati et al., 2011; Wang et al., 2014; Niu et al., 2021). In *Arabidopsis*, there are 17 known members of the laccase family, which are classified into six groups (McCaig et al., 2005; Hoegger et al., 2006; Turlapati et al., 2011), of which eight are expressed in stems (Berthet et al., 2011; Zhao et al., 2013). *LAC2*, *LAC8*, and *LAC15* play roles in root elongation, early flowering, and changes in seed colour, respectively (Cai et al., 2006). Only four laccase genes, namely *LAC4*, *LAC11*, *LAC15*, and *LAC17*, have been reported to participate in lignin biosynthesis (Liang et al., 2006; Wang J. et al., 2008; Berthet et al., 2011; Zhao et al., 2013). Three double mutants, *lac4lac17, lac4lac11*, and *lac11 lac17*, showed mild reduction in lignin biosynthesis, whereas the triple mutant *lac4lac11lac17* almost completely abolished lignin deposition, leading to severe plant growth arrest (Zhao et al., 2013). In these mutants, although lignin content was observed to have remarkably decreased, the amount of S-lignin barely changed, indicating that *LAC4*, *LAC11*, and *LAC17* act in G-lignin biosynthesis (Berthet et al., 2011). Laccases have been documented in lignin biosynthesis, but definitive physiological functions and regulation of plant laccases by microRNAs (miRNAs) remain to be elucidated.

miRNAs, endogenous small non-coding RNAs (21–24 nucleotides), play crucial roles in the regulation of gene expression (Vaucheret, 2006; Filipowicz et al., 2008; Brodersen and Voinnet, 2009). Several miRNAs, viz. miR397, miR408, and miR857, have been validated to target plant laccase genes, thus showing potential for lignin engineering (Sunkar and Zhu, 2004; Li et al., 2010). miR397-directed cleavage of laccase transcripts has been observed and acts as a negative regulator of lignin content in *Arabidopsis* (Abdel-Ghany and Pilon, 2008; Yamasaki et al., 2009; Wang et al., 2014) and *Populus trichocarpa* (Lu et al., 2008). Rice miR397 negatively regulates *OsLAC* transcriptional levels, resulting in increased grain size and branch number (Zhang et al., 2013). However, miRNAs that regulate LACs in plant lignin biosynthesis need to be investigated.

Plants have evolved multiple defence mechanisms against pathogenic infections (Miedes et al., 2014; Zhang et al., 2017). Phytopathogenic fungi can secrete various hydrolases to degrade most components of plant cell walls, providing carbon sources for pathogen growth and triggering multiple plant defence responses (Tayi et al., 2016). However, lignin is very difficult to degrade and is also regarded as a component of the defence response in plants (Miedes et al., 2014; Vanholme et al., 2019). Accumulating evidence has shown that lignin is a defensive physical/chemical barrier that restricts pathogen growth (Bonello and Blodgett, 2003; Zhang et al., 2017). These studies mainly manipulated lignin content and composition by regulating the monolignol pathway consisting of 11 enzymatic steps (Vanholme et al., 2013). However, it cannot distinguish between cell wall lignification and defence-induced lignification in the resistance against (hemi)biotrophic pathogens (Bhuiyan et al., 2009). Defence-induced lignification is a conserved basal defence mechanism in the plant immune response against (hemi)biotrophic pathogens in a wide range of plant species and is regarded as a biochemical marker of an activated immune response (Menden et al., 2007; Bhuiyan et al., 2009; Adams-Phillips et al., 2010; Kishi-Kaboshi et al., 2010). For example, in *Arabidopsis* and tobacco (*Nicotiana tabacum*), phenylalanine ammonia-lyase (*PAL*) has been verified as a basal immunity against the hemibiotrophic bacterial pathogen *Pseudomonas syringae* and biotrophic viral pathogen tobacco mosaic virus, respectively (Huang et al., 2010). Recently, *GhLAC1* overexpression has been identified to increase lignification and promote jasmonic acid (JA) biosynthesis, balancing the JA–SA defence response, indicating that GhLAC1 is a broad-spectrum biotic stress tolerance protein (Hu et al., 2018). Thus, cotton LACs functioning in lignin biosynthesis remain unclear, and it is also vital to evaluate whether *GhLACs* participate in plant resistance against *V. dahliae*.

Cotton is an important cash crop for its natural fibres, oil, and feed worldwide. However, cotton production is affected by the damage of verticillium wilt, which is caused by *V. dahliae* (Bolek et al., 2005; Cai et al., 2009; Zhang et al., 2019). In this study, we characterised roles of a cotton laccase gene, *GhLAC4*, in lignin biosynthesis and plant resistance against *V. dahliae* infection via the regulation of ghr-miR397. *GhLAC4* and ghr-miR397 were induced by *V. dahliae* infection. The ghr-miR397-*GhLAC4* module was found to mediate both basal lignin and defence-induced lignin biosynthesis, protecting plants from pathogenic invasions.

## Materials and Methods

### Plant Growth and Hormones Treatment

Seeds of *Gossypium hirsutum* cv. Jihe713 were germinated and grown in the greenhouse for approximately 2 weeks at 28°C/25°C with a 16 h light/8 h dark photoperiod. In order to detect cotton gene expression pattern, germinating seeds were transferred in the box containing Hoagland’s nutrient solution for seedling growth. Seedlings grown in nutrient soil were used to conduct virus-induced gene silencing (VIGS).

*Nicotiana benthamiana* plants were grown in pots at 23°C ingrowth chambers under a 16 h light/8 h dark photoperiod with 60% humidity.

### Phylogenetic Analysis

The LACs of *G. hirsutum* and *Arabidopsis thaliana* were obtained from cotton database^1^ and TAIR database^2^, respectively. Amino acid sequence alignment and phylogenetic analysis were performed on Clustal X2.1 and Mega 7.0, respectively.

### Extraction of RNA and Real-Time Quantitative PCR

A sample of cotton tissue was taken and ground quickly with liquid nitrogen in a sterilised mortar. The ground sample was dispensed into an enzyme-free centrifuge tube, and then total RNA was isolated from the cotton tissue using TRIzol reagent (Invitrogen, Carlsbad, CA, United States). Reverse transcription of RNA to synthesise cDNA by using EasyScript One-Step gDNA Removal and cDNA Synthesis SuperMix Kit (Qinke Biotech, Beijing, China). Then SYBR Green real time PCR were performed using commercial kits (Qinke Biotech, Beijing) according to the manufacture’s protocol. The miRNA primer design, first-strand cDNA synthesis and qPCR analysis all refer to the method of Varkonyi-Gasic et al. (2007). The miRNA and other genes expression was normalised to *U6* and *UBQ7*, respectively. The relative expression level was shown using the 2^–ΔΔCT^ method. Each sample mean with standard derivation (SD) of the relative expression level come from measuring values containing at least 4 treated or untreated plants with 3 experiment repeats.

### Gene Isolation, Vector Construction and Agroinfiltration of Plants

For the virus-induced gene silencing (VIGS) analysis, *tobacco rattle virus* (TRV)-based vectors, including pTRV1 (pYL192), pTRV2 (pYL156), and pTRV2e were used in this study. TRV:PDS (phytoene desaturase, PDS) was employed as a positive control vector in the silenced plants, which had been previously reported by Pang et al. (2013). The construction of the TRV-related vectors was performed as described by Liu et al. (2004) and Zhao et al. (2020). Briefly, to overexpress ghr-miR397, pre-miR397 sequence was isolated and was inserted into pTRV2e plasmid by restriction enzyme digestion sites *Xba*I and *Bam*HI under control of sgp promoter, named OE-miR397 (Supplementary Figure 1A). To generate the TRV:STTM397 vector for silencing ghr-miR397, a small tandem target mimic (STTM) sequence of ghr-miR397 containing two imperfect ghr-miR397 binding sites separated by a 48-bp spacer with the restriction enzyme sites *Kpn*I and *Bam*HI at the 5′ and 3′ ends, respectively, was designed and inserted into the pTRV2e vector (Supplementary Figure 1B). A *GhLAC4* specific fragment was isolated and inserted into pTRV2, and the resulting vector was designated TRV:GhLAC4.

The *GhLAC4* and *GhLAC4* mutant (*GhLAC4*^*mu*^, mutated in target sequence of ghr-miR397) were fused with the *GUS* gene of the pBI121 vector to generate the GhLAC4:GUS and GhLAC4^mu^:GUS vector, respectively. The precursor sequence of miR397 was inserted into pCAMBIA1300 and driven by 35S promoter, to generate pCAMBIA1300-miR397. All the plasmids were transformed into *Agrobacterium tumefaciens* strain GV3101 using electroporation. *A. tumefaciens* containing the indicated vectors was cultured in LB medium with 50 μg/mL kanamycin, 50 μg/mL gentamicin and 50 μg/mL rifampicin at 28°C overnight. The Agrobacterium cells were collected and then resuspended in MMA solution (10 mM N-morpholino ethanesulfonic acid, 10 mM MgCl_2_, and 200 mM acetosyringone, OD600 = 1.2). The Agrobacterium cells containing the above vectors were equally mixed with pYL192 and incubated at room temperature in darkness for 2 h. Finally, plants were agroinfiltrated as previously described by Tang et al. (2019). All the primers associated with vector construction are listed in Supplementary Table 1. The same experiment was repeated three times.

### GUS Histochemical Staining

The GUS transient expression in the tobacco leaves, histochemical staining and activity quantified was performed as described by Hu et al. (2020b). In brief, the leaves were placed in pre-cooled 95% acetone on ice overnight. Then the leaves washed by 100 mM PBS buffer (pH = 7.0) and added the GUS staining solution [1 mM K_3_Fe(CN)_6_, 1 mM K_4_Fe(CN)_6_, 10 mM Na_2_EDTA (pH = 8.0), 1 mg/mL *X*-Gluc and 1 ml/L Triton X-100] 37°C for overnight. The leaves were washed with 75% alcohol, photos were taken until the green leaves completely fade.

### Histochemical Staining, Determination of Lignin Content and Composition

To observe the xylem development and lignin deposition, Wiesner reagent was used for histochemical staining (Xu et al., 2011; Tang et al., 2019). In brief, more than 3 cotton first internodes were sliced by hand, which were Wiesner stained with phloroglucinol-HCl (Prolabo). To distinguish the G-lignin unit from lignin components, Maüle staining was performed (Berthet et al., 2011). Briefly, slices of cotton first internodes were incubated in 5% KMnO_4_ for 7 min, and washed with ddH_2_O, following acidification with 20% HCl for 5 min. Then acidified samples washed with ddH_2_O were incubated with 29% ammonia for 5 min. The stained slices were observed and photographed using a stereomicroscope (DM2500; Leica, Germany).

The lignin contents of the first internodes from the different plants were measured by the Klason method as previously described (Abdullah et al., 2006). And the composition of lignin in the first internodes of cotton was determined by thioacidolysis (Zhao et al., 2010). Lignin-derived compounds were identified by gas chromatography mass spectrometry (GC/MS), and its trimethylsilyl derivatives were quantified by GC. GC/MS was performed on Aglient 7890A GC-5975C MS (G3172A, United States). The same experiment was repeated three times.

### Culture of *V. dahliae* and Treatment of Infecting Cotton

A tube of *V. dahliae* spore solution was taken from the refrigerator at −80°C and spread on a PDA plate, which was incubated at 28°C for 2–3 days. An appropriate amount of mycelium from the plate was scraped to inoculate it into Czapek–Dox Medium to culture at 28°C and 200 r/min for 4–5 days. For inoculum preparation, fungal cultures were filtered with gauze, and the number of spores were counted with a hemocytometer under a microscope. It was diluted with distilled water to a concentration of 1 × 10^6^ conidia/mL for later inoculation.

When the cotton plants had grown for about 3–4 weeks in soil pots with 3–4 true leaves. Plant roots free of soil were soaked in a treatment solution with a concentration of 1 × 10^6^ conidia/mL *V. dahliae* V991 for 2 min and covered with plastic overnight. Then, the treated plants were planted in soil pots and transferred to a light incubator to culture. Mock treatment with fungus-free solution were performed as control group. The similar experiment was conducted to inoculate seedlings grown in nutrition solution. The roots of seedlings were dipped into the pathogen spore solution for inoculation. Each sample contained 30 plants with inoculation or mock treatment. The same experiment was performed three times.

### Analysis of Disease Symptom and Disease Index

Twenty-one days’ post-inoculation (dpi), the disease symptoms such as leaf yellowing, wilting, shedding and even death appeared in treated plants. The disease plant rate and DI of the inoculated plants were calculated. The DI statistics including disease grade refers to the method reported by Wang Y.Q. et al. (2008).

DI = Σ (number of infected plants × disease grade)/(total number of plants × 4) × 100.

### Measure of Pathogenic Biomass in Treated Plants

To observe the degree of browning of the xylem, the first internodes of 21 dpi plants were taken and cut by hand. And the first internodes of the inoculated plants were then cut into fragments to carry out restoring culture according to the method reported by Tang et al. (2019). In brief, the sterilised internodes were cut into 0.5–1 cm fragments and placed on the PDA solid medium, which were incubated at 28°C in the dark for 7 days for recovering growth of *V. dahliae*.

To further investigate pathogenic biomass in the inoculated cotton plants, DNA of stems in the infected plants was extracted to quantify the content of *V. dahliae* by qPCR analysis. The specific primer *V. dahliae* β*-tubulin-F/R* of was designed by Primer Premier 5.0 software (Supplementary Table 1). The relative content of genes was analysed using *GhUBQ7* as an internal reference for sample homogenization.

### Measurement of Phytohormones

To measure the endogenous concentrations of JA and SA, root samples of about 100–200 mg were homogenised twice with 80% (V/V) cold methanol and shaken overnight in 4°C darkness. Dissolution, filtration, storage and quantification of combined extracts are described by Sun et al. (2014). SA and JA content were measured by HPLC-MS/MS performed by AB SCIEX QTRAP 4500 system (AB SCIEX, Foster City, CA, United States) as described by Zhou et al. (2018). The experiment was repeated 3 times with more than 3 seedlings in the sample.

### Cellulolysis Assays

The extract-free stems were employed to analyse the lignin protection to cell wall from degradation by cellulase as previously described by Zhang et al. (2017). About 20 mg sample was soaked in 20 mL 0.05 M sodium acetate buffer (pH 4.7) supplemented with 2 mg/mL commercial cellulase (cellulase Onozuka-R10; Serva). After incubating at 37°C with magnetic stirring for 4 days, the samples were filtered and weighed again. The similar experiment was performed by using filtering solution of *V. dahliae* culture instead of cellulase. The Glc released into the filtrate by cellulase degradation was quantified using the BioMerieux Glucose RTU kit (BioMerieux, Marcy-l’Étoile, France) as previously described by Berthet et al. (2011).

## Results

### Ghr-miR397 Expression Inhibition by *V. dahliae* Infection

**FIGURE 1:**
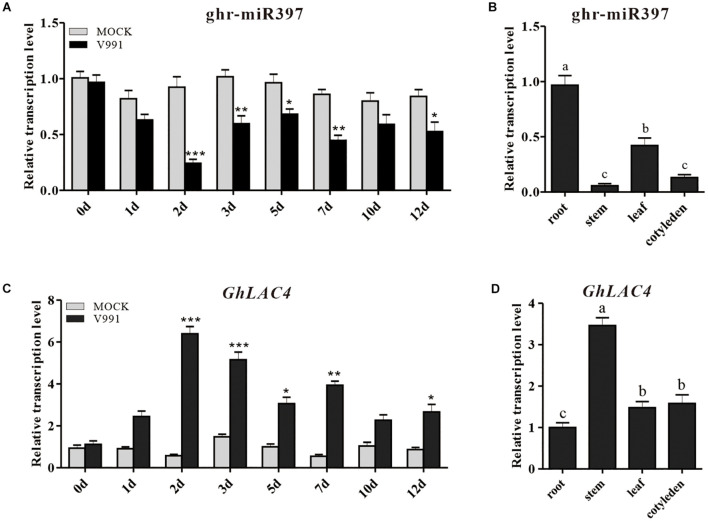
Expression pattern analysis of ghr-miR397 and *GhLAC4*. **(A)** The ghr-miR397 accumulation under *Verticillium dahliae* infection and mock treatment. **(B)** The expression profile of ghr-miR397 in various cotton tissues. **(C)** Expression profile of *GhLAC4* in the time course of cotton roots infected with *V. dahliae*. **(D)** Expression analysis of *GhLAC4* in various cotton tissues. The abundance values of ghr-miR397 and *GhLAC4* in cotton roots were arbitrarily assigned as ‘1.’ Mean with SD comes from triple repeats experiments. Significant differences in **(A,C)** were determined using Student’s *t*-test (*P < 0.05, **P < 0.01, ***P < 0.001). Different letters in **(B,D)** indicate significant differences (*P* < 0.05) based on Tukey’s HSD test.

In our previous miRNA data on *G. hirsutum* root response to *V. dahliae* infection, a new miRNA (novel miR_D05_24647) with different expression change compared to the mock control was identified (Hu et al., 2020a). The novel miR_D05_24647 was highly homologous with *Linum usitatissimum* miR397a, Brachypodium distachyon miR397b-5p, and *A. thaliana* miR397a as revealed by the homologous alignment analysis in miRBase (Supplementary Figure 2A); hence, it was named as ghr-miR397. The 21 nucleotides long ghr-miR397 is produced at the 5′-terminus of pre-miR397, which has a typical stem-loop structure (Supplementary Figure 2B). To further confirm the induced expression of ghr-miR397, cotton seedlings were inoculated with *V. dahliae* strain V991 using the root dipping method. qPCR analysis showed that the expression of ghr-miR397 was inhibited in inoculated plants compared to that in the mock plants. The ghr-miR397 accumulation was significantly lower at 2, 3, and 7 dpi, suggesting that ghr-miR397 may participate in plant disease resistance (Figure 1A). Additionally, the analysis of cotton tissue expression profiles showed that ghr-miR397 was constitutively expressed in roots, leaves, cotyledons and stems, while predominantly expressed in roots (Figure 1B).

### Analysis of GhLAC4 Characteristics and Expression Response

In our previous database, ghr-miR397 was identified to regulate 6 target genes namely Gh_A03G0417, Gh_D03G1128, Gh_A03G2084, Gh_D13G2373, Gh_A13G2215 and Gh_D13G2524, out of which, Gh_A03G0417 and Gh_D03G1128 showed remarkable upregulation expression under *V. dahliae* treatment (Supplementary Figure 3) and located on the A and D subgenomes of *G. hirsutum* (Hu et al., 2020b). Therefore, two target genes were suffered to further research. The corresponding two encoded proteins showed high identities in nucleotides and amino acids (Supplementary Figures 4, 5), consequently regarded as a gene, predicted to be a laccase. Eighty-two *G. hirsutum* laccases and 17 *Arabidopsis* laccases were used to carry out a phylogenetic tree analysis and were classified into 6 groups. Gh_A03G0417 (GhLAC4-9) and Gh_D03G1128 (GhLAC4-8) were clustered with AtLAC4 that belonged to Group 2 (Supplementary Figure 6). Thus, both were named *GhLAC4*, which encodes a 556 amino acid peptide (Supplementary Figure 2C). In *Arabidopsis*, there are only four laccase genes, namely *LAC4*, *LAC11*, *LAC15*, and *LAC17*, which participate in lignin biosynthesis (Miedes et al., 2014; Zhang et al., 2019). Alignment analysis of GhLAC4 and four lignin-biosynthesis-related laccases showed that GhLAC4 was highly similar to AtLAC4, AtLAC11, AtLAC15, and AtLAC17, showing 75.81, 59.86, 40.70, and 54.23% sequence identity, respectively (Supplementary Figure 7). These laccases contain three oxidase domains and four typical laccase copper ion binding domains, indicating that they may be functionally conserved in lignin biosynthesis.

To characterise the role of *GhLAC4* in plant resistance to *V. dahliae*, its expression profiles were examined. *GhLAC4* expression levels in plants treated with *V. dahliae* and mock were subsequently analysed by qPCR. Results showed that *GhLAC4* expression significantly increased in plants inoculated with *V. dahliae* compared to the mock control (Figure 1C), showing a negative correlation with the expression of ghr-miR397 (Figure 1A). *GhLAC4* was found to be constitutively expressed in cotton roots, stems, leaves, and cotyledons, and was preferentially expressed in stems; the expression pattern showed a negative correlation with ghr-miR397 tissue expression levels (Figure 1D).

### Directed Cleavage of *GhLAC4* Transcript by ghr-miR397

We then evaluated whether and how ghr-miR397 regulates the post-transcriptional expression level *GhLAC4*. There were 19 complementary pairs between ghr-miR397 and *GhLAC4* mRNA target sequences at 666–684 nt (Supplementary Figure 2C). In our previous degradome sequencing, ghr-miR397-directed cleavage was found to occur at 678th nucleotide of *GhLAC4* mRNA between C and U bases, and the cleavage site was between 10th and 11th nucleotides of ghr-miR397 (Supplementary Figure 2D).

**FIGURE 2:**
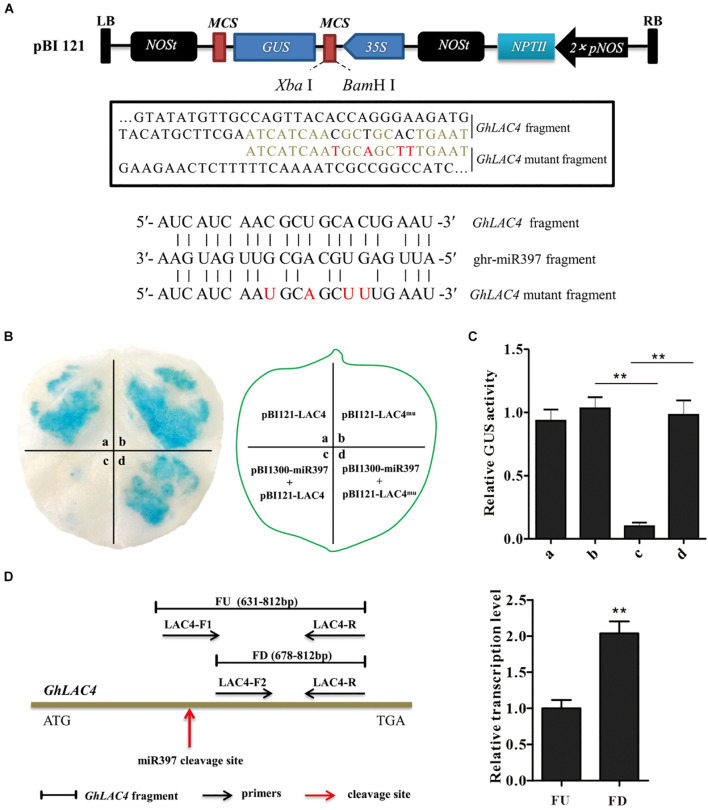
ghr-miR397 regulates the expression of *GhLAC4* through post-transcriptional processing. **(A)** Schematic diagram of pBI121-LAC4 and pBI121-LAC4 mutant. Red letters represent the mutated bases. **(B)** GUS tissue staining leaf spots (left panel) infiltrated with different vectors as indicated (right panel). **(C)** Quantitative analysis of GUS activities of different leaf sports corresponding to **(B)** with the 4-MU assay. The GUS activity value of at a site is arbitrarily designated as ‘1.’ **(D)** qPCR analysis of ghr-miR397-directed cleavage of *GhLAC4* mRNA. Illustration of the primers designed (left panel), fragments FD and FU represent PCR amplification products located downstream of and containing the cleavage site, respectively (right panel). Mean with SD comes from triple repeats experiments. Significant differences in **(C,D)** were determined using Student’s *t*-test (**P < 0.01).

To verify ghr-miR397-directed *GhLAC4* mRNA cleavage, ghr-miR397 was introduced into a plant overexpression vector as a driver vector (pCAMBIA1300-miR397), and either *GhLAC4* or target sequence-mutated *GhLAC4* (GhLAC4^mu^) was fused into GUS protein and converted into effector vectors GhLAC4:GUS or GhLAC4^mu^:GUS (Figure 2A), respectively. The vectors were transformed into *A. tumefaciens* via electroporation, which was then injected into tobacco leaves for transient expression. As shown in Figure 2B, the leaf spots agroinfiltrated with GhLAC4:GUS or GhLAC4^mu^:GUS were stained as normal blue under GUS staining 48 h after injection. Agroinfiltrated leaf spots with the combination of pCAMBIA1300-miR397 and GhLAC4:GUS showed lighter blue colour, while those treated with pCAMBIA1300-miR397 and GhLAC4^mu^:GUS still exhibited normal blue colour, indicating that ghr-miR397 directedly cleaved *GhLAC4* mRNA. In line with these results, analysis of GUS enzyme activity demonstrated that ghr-miR397 could inhibit *GhLAC4* expression (Figure 2C).

To further confirm ghr-miR397-directed *GhLAC4* cleavage, two positive primers (LAC4F1 and LAC4F2) upstream and downstream of the cleavage site were designed (Figure 2D, left panel). After the amplification efficiency of the two positive primers was corrected, the amounts of the FD amplified fragment downstream of the cleavage site were approximately twofold higher of the FU amplified fragment containing the cleavage site (Figure 2D, right panel). Suggesting that part of *GhLAC4* mRNA might be cleaved at a specific site between two positive primers (LAC4F1 and LAC4F2). These results indicated that ghr-miR397-directed *GhLAC4* mRNA cleavage occurred in cells.

### Ghr-miR397 Negatively Regulates Plant Resistance Against *V. dahliae*

**FIGURE 3:**
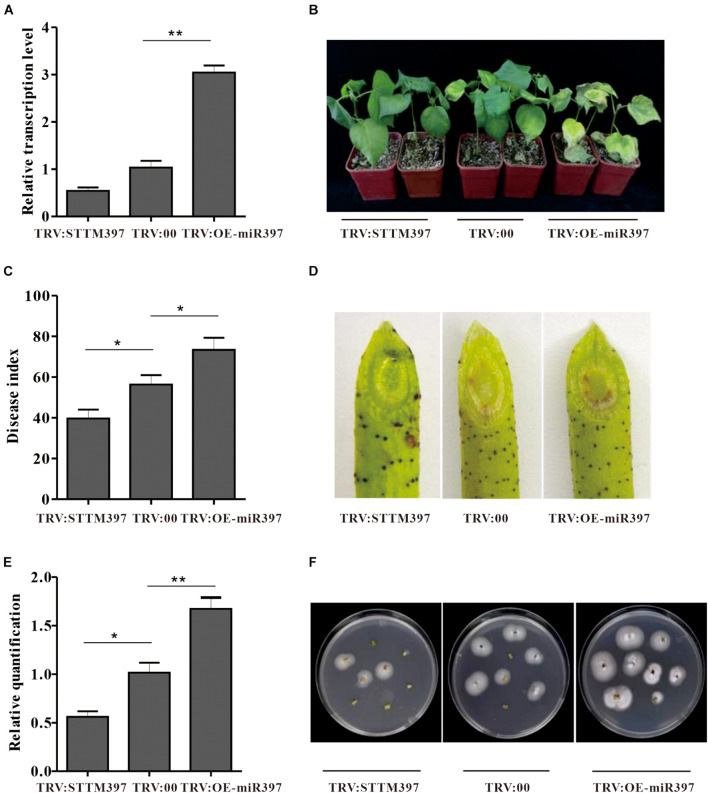
The miR397 negatively regulated plant resistance against *V. dahliae*. **(A)** Relative expression levels of ghr-miR397 in the TRV:STTM397 and TRV:OE-miR397 plants compared to the TRV:00 plants. The value of ghr-miR397 abundance at the TRV:00 plants were arbitrarily designated as ‘1.’ **(B)** Disease symptom of the TRV:STTM397, TRV:OE-miR397, and TRV:00 plants inoculated with *V. dahliae*. **(C)** The disease index of plants 21 dpi. **(D)** The brown colour of vascular tissue of plants 21 dpi. **(E)** The *V. dahliae* biomass in the infested stems by qPCR. The DNA content of *V. dahliae* is relatively quantified through comparison of fungal β*-tubulin* gene and cotton *UBQ7* gene. **(F)** Fungus recovery assay 21 dpi. The stem of the TRV:STTM397, TRV:OE-miR397, and TRV:00 plants was cut into fragments placed on PDA media. Photos were taken 5 days after plating. Mean with SD comes from triple repeats experiments. Significant differences in **(A,C,E)** were determined using Student’s *t*-test (*P < 0.05, **P < 0.01).

To elucidate ghr-miR397 function in plant resistance against *V. dahliae*, ghr-miR397-silenced plants were generated through the silencing system using tobacco rattle virus (TRV) and short-tandem target mimic (STTM) technology. When the phytoene desaturase (PDS)-silenced plants showed a photo-bleaching phenotype as a marker (Supplementary Figure 8), ghr-miR397 accumulation in TRV:STTM397 plants was analysed by qPCR analysis. Compared to the control plants agroinfiltrated with empty vectors (TRV:00), the expression levels of ghr-miR397 in TRV:STTM397 plants were significantly reduced by approximately 50% (Figure 3A); and the relative expression of *GhLAC4* significantly increased in TRV:STTM397 plants (Supplementary Figure 9). These plants were subsequently inoculated with *V. dahliae* by root-dipped method. At 21 dpi, the disease symptoms of ghr-miR397-silenced plants was milder with less wilt and yellow leaves compared to TRV:00 plants (Figure 3B). The disease index of TRV:STTM397 plants was significantly lower than that of the control (40 vs. 57) (Figure 3C). The brown colour of vascular tissue in oblique sections of the stems was remarkably lighter in TRV:STTM397 plants than in TRV:00 plants, suggesting that ghr-miR397 knockdown increased plant resistance against *V. dahliae* (Figure 3D). The analysis of fungal recovery from stem sections and fungal biomass in stems confirmed that ghr-miR397 knockdown significantly repressed fungal growth in TRV:STTM397 plants compared to the TRV:00 plants (Figures 3E,F). These results showed that ghr-miR397 could negatively regulate plant resistance against *V. dahliae*.

To further verify ghr-miR397 function in plant resistance to *V. dahliae*, overexpression of ghr-miR397 plants (TRV:OE-miR397) was achieved through the TRV overexpression system. The mean expression level of TRV:OE-miR397 plants was approximately 3-folds higher than that of the TRV:00 plants (Figure 3A), the relative expression of *GhLAC4* in TRV:OE-miR397 plants significantly reduced (Supplementary Figure 9). Supporting the notion that ghr-miR397 negatively regulates plant resistance, TRV:OE-miR397 plants exhibited higher susceptibility to *V. dahliae* than TRV:00 plants, with more wilt and yellow leaves (Figure 3B), higher disease index (Figure 3C), more fungal biomass indicated by heavier browning colour, more fungal recovery stems, and higher fungus in number (Figures 3D–F).

### Cotton miR397-LAC4 Module Regulates Plant Resistance Against *V. dahliae*

**FIGURE 4:**
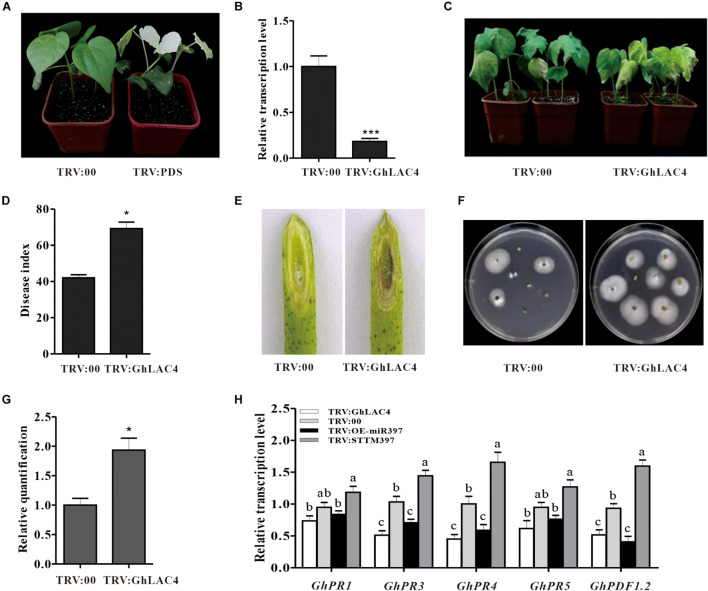
The cotton miR397-LAC4 module mediated plant resistance to *V. dahliae.* **(A)** Photo-bleaching leaves of *GhPDS*-silenced plants. **(B)** Relative expression levels of *GhLAC4* in the TRV: GhLAC4 plants compared to the TRV:00 plants. **(C)** Disease symptom of the TRV: GhLAC4 and TRV:00 plants inoculated with *V. dahliae*. **(D)** The disease index of the treated plants 21 dpi. **(E)** The brown colour of vascular tissue of plants 21 dpi. **(F)** Fungus recovery from 21 dpi stem fragments cultured for 5 days. **(G)** The *V. dahliae* biomass in the infested stems by qPCR. The DNA content of *V. dahliae* is relatively quantified through comparison of fungal β*-tubulin* gene and cotton *UBQ7* gene. **(H)** Expression levels of the defence-related genes in TRV:STTM397, TRV:OE-miR397, TRV:GhLAC4, and the control plants inoculated with *V. dahliae. GhUBQ7* was an internal reference gene. Mean with SD comes from triple repeats experiments. Significant differences in **(B,D,G)** were determined using Student’s *t*-test (*P < 0.05, ***P < 0.001). Different letters in **(H)** indicate significant difference (*P* < 0.05) based on Tukey’s HSD test.

To elucidate the function of the cotton miR397-LAC4 module in plant response to *V. dahliae* infection, *GhLAC4* knockdown plants (TRV:GhLAC4) were generated using the VIGS method. It is noted that the specific fragment of *GhLAC4* was chosen according to nucleotide assignment with two closer identity genes, *GhLAC11* and *GhLAC22* (Supplementary Figures 10A,B). When *PDS*-silenced plants exhibited a photobleaching phenotype (Figure 4A), the expression level of *GhLAC4* in silenced plants was examined by qPCR. Compared to control plants (TRV:00), *GhLAC4* expression level in *GhLAC4*-silenced plants was significantly decreased to 0.2-fold (20%) level of that observed for the control (Figure 4B). And expression levels of *GhLAC11* and *GhLAC22* in *GhLAC4*-silenced plants were similar as that in TRV:00 (Supplementary Figure 10C). TRV:GhLAC4 and TRV:00 plants were inoculated with *V. dahliae*. At 21 dpi, GhLAC4-silenced plants showed more susceptibility to pathogenic infection with more yellow and wilt leaves than TRV:00 plants (Figure 4C). The disease index of TRV:GhLAC4 plants was significantly higher than that of TRV:00 plants (69 vs. 42) (Figure 4D). Supporting these results, compared to the control, the brown colour of vascular tissue was darker (Figure 4E), more colonies were recovered from the *GhLAC4*-silenced stem fragments (Figure 4F), and more pathogen biomass was observed in these plants (Figure 4G). These results showed that silencing *GhLAC4* reduced plant resistance to *V. dahliae*.

**FIGURE 5:**
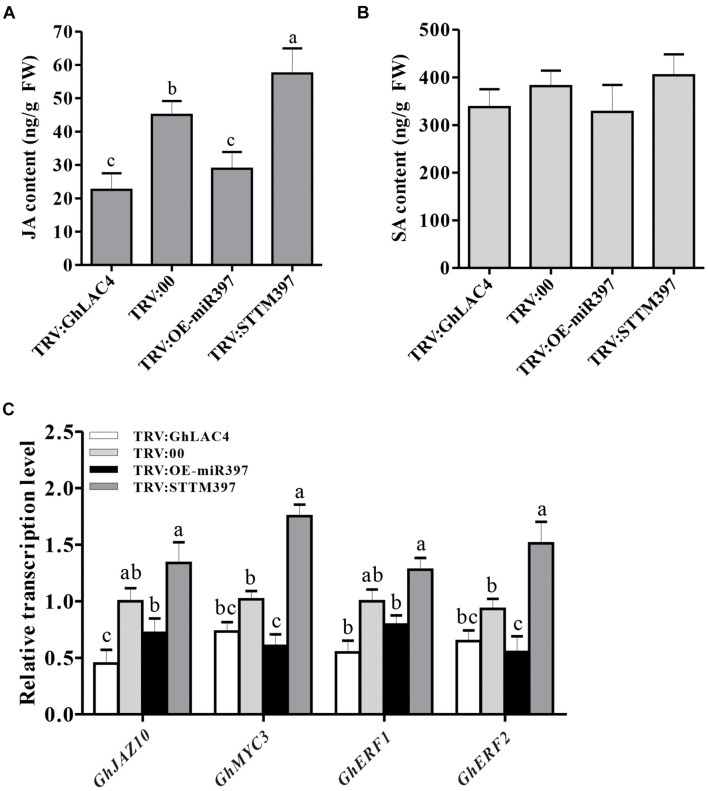
Measurement of JA and SA content in the plants inoculated with *V. dahliae*. **(A)** JA contents in TRV:STTM397, TRV:OE-miR397, TRV:GhLAC4, and the control plants inoculated with *V. dahliae*. **(B)** SA contents in TRV:STTM397, TRV:OE-miR397, TRV:GhLAC4, and the control plants inoculated with *V. dahliae*. **(C)** Expression analysis of JA signalling pathway-related genes with *V. dahliae*. *GhUBQ7* was an internal reference gene. Mean with SD comes from triple repeats experiments. Different letters in **(A–C)** indicate significant difference (*P* < 0.05) based on Tukey’s HSD test.

Previous studies have documented that LACs participate in plant response to pathogens by manipulating the SA and JA synthesis (Hu et al., 2018). To investigate whether cotton miR397-LAC4 module participating in plant resistance to *V. dahliae* implicates SA and/or JA pathway, the expression levels of *GhPR1*, *GhPR3*, *GhPR4*, *GhPR5*, and *GhPDF1.2* were measured in VIGS plants under fungal treatment. The *GhPR1* and *GhPR5* expression levels were observed to have slightly decreased in both TRV:GhLAC4 and TRV:OE-miR397 plants compared to the control, whereas that in TRV:STTM397 plants was found to have slightly increased; however, these differences did not reach a significant level as revealed by the statistical analysis (Figure 4H), which indicates that the regulation of plant resistance by ghr-miR397-GhLAC4 module is not mediated via the SA pathway. On the other hand, the expression levels of *GhPR3*, *GhPR4*, and *GhPDF1.2* in both TRV:GhLAC4 and TRV:OE-miR397 plants were significantly lower than those in the control plants, while those in TRV:STTM397 plants were found to have significantly increased (Figure 4H), suggesting that the participation of ghr-miR397-GhLAC4 module in plant resistance to *V. dahliae* could implicate the JA biosynthesis as well as *GhLAC1* reported by Hu et al. (2018). To verify this conclusion, the JA and SA contents in TRV:GhLAC4, TRV:STTM397, and TRV:OE-miR397 plants were measured by HPLC. The results showed that JA level in TRV:STTM397 was significantly higher compared to the control, while those in TRV:GhLAC4 and TRV:OE-miR397 plants significantly decreased (Figure 5A). However, SA contents show no significant differences between the control and in TRV:GhLAC4, TRV:OE-miR397, and TRV:STTM397 plants (Figure 5B).

To determine whether the ghr-miR397-GhLAC4 module manipulating JA biosynthesis affects the JA signalling pathway, we examined the expression response of JA-related genes in VIGS plants inoculated with *V. dahliae*. The expression levels of *GhJAZ10*, *GhMYC2*, *GhMYC3*, and *GhERF1* were significantly reduced in TRV:GhLAC4 and TRV:OE-miR397 plants, whereas they were found to have significantly increased in TRV:STTM397 plants, compared to the control plants (Figure 5C). These results demonstrated that the ghr-miR397-GhLAC4 module participates in plant resistance against *V. dahliae* partially through implicating JA biosynthesis.

### *GhLAC4* Participates in Plant Lignin Biosynthesis by Regulation of ghr-miR397

**FIGURE 6:**
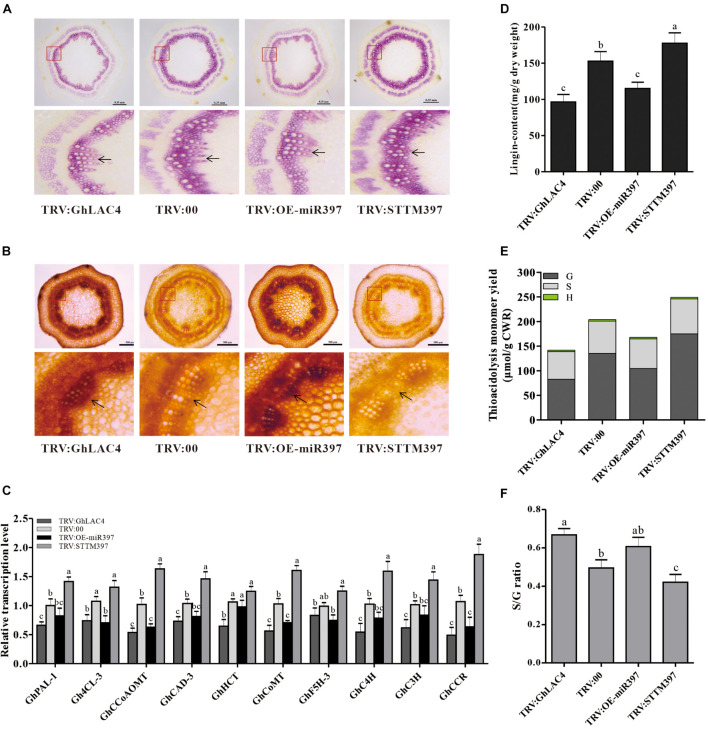
Analysis of expression levels of lignin biosynthesis-related genes and lignin content and composition. **(A,B)** Stem cross slices stained with phloroglucinol-HCl **(A)** and Maüle staining method **(B)** in TRV:STTM397, TRV:OE-miR397, TRV:GhLAC4, and the control plants. The red boxes in up panels are enlarged as shown in down panels; the black arrow points to lignin fibres. Scale bars in **(A)** is 0.35 mm, **(B)** scale bars is 500 μm. **(C)** The relative expression levels of lignin biosynthesis-related genes in TRV:STTM397, TRV:OE-miR397, TRV:GhLAC4, and the control plants. **(D)** Analysis of Klason lignin content in TRV:STTM397, TRV:OE-miR397, TRV:GhLAC4, and the control plants. **(E)** Determination of the main H, G, and S thioacidolysis monomers released by the lignins of extract-free mature stems of laccase in TRV:STTM397, TRV:OE-miR397, TRV:GhLAC4, and the control plants. **(F)** S/G molar ratio in in TRV:STTM397, TRV:OE-miR397, TRV:GhLAC4, and the control plants. Mean with SD comes from triple repeats experiments. Different letters in **(C,D,F)** indicate significant difference (*P* < 0.05) based on Tukey’s HSD test.

Laccase can polymerise monolignols into lignin to deposit in the cell wall, which is an important barrier for pathogen invasion (Barros-Rios et al., 2011; Zhang et al., 2017, 2019). To explore whether *GhLAC4* participates in lignin biosynthesis in the regulation of ghr-miR397, lignin levels were tested in VIGS plants by phloroglucinol HCl staining. As shown in Figure 6A, intensities of the red staining in TRV:GhLAC4 and TRV:OE-miR397 plants were lighter than those in TRV:00 plants, while the red colour in TRV:STTM397 plants was darker (Figure 6A), verifying that *GhLAC4* regulated by ghr-miR397 participates in lignin biosynthesis. Analysis of Maüle staining in the stem slices revealed that the xylem parts in both TRV:GhLAC4 and TRV:OE-miR397 plants showed a darker brown colour than that in TRV:00 plants, whereas that in TRV:STTM397 plants was lighter (yellow colour, Figure 6B). Yellow colour in Maüle staining analysis is considered to indicated the presence of G-lignin, which is reportedly the main lignin that participates in plant resistance to pathogen invasion (Chezem et al., 2017; Zhang et al., 2019). In line with these results, the expression levels of lignin biosynthesis-related genes and lignin content were measured in VIGS plants. The levels of 10 lignin biosynthesis-related genes, namely *GhPAL*, *Gh4CL*, *GhCCoAOMT*, *GhCAD-3*, *GhHCT*, *GhCoMT*, *GhC4H*, *GhC3H* and *GhCCR*, and *Gh5FH-3*, located in the upstream of *GhLAC4* were found to have significantly decreased in both TRV:GhLAC4 and TRV:OE-miR397 plants than those in TRV:00 plants; however, these levels were found to have significantly increased in TRV:STTM397 plants (Figure 6C). The results suggested that under the weakening of *GhLAC4* function which possibly leads to monolignols (substrate) accumulation, plant feedback decreased expression levels of the upstream lignin synthesis genes, and *vice versa*, which is consistent with the report in *Arabidopsis* (Zhao et al., 2013; Tang et al., 2019). The lignin content measured by the Klason method in both TRV:GhLAC4 and TRV:OE-miR397 plants was significantly lower than that in TRV:00 plants, whereas that in TRV:STTM397 plants was significantly higher (Figure 6D). GC/MS analysis showed that G-lignin content in TRV:STTM397 plants was significantly higher than that in the control, whereas that in TRV:GhLAC4 and TRV:OE-miR397 plants was significantly lower (Figure 6E). The S-lignin/G-lignin ratio in TRV:STTM397 plants was also lower than that in the control, while this ratio in TRV:GhLAC4 and TRV:OE-miR397 plants was observed to have been heighten (Figure 6F). A similar result stating that *Arabidopsis LAC4*, *LAC11*, and *LAC17* participate in G-lignin, not S-lignin, biosynthesis, has been reported earlier (Berthet et al., 2011). These results demonstrated that *GhLAC4* regulated by ghr-miR397 participated in plant lignin biosynthesis, especially G-lignin biosynthesis, facilitating cotton plant resistance against *V. dahliae.*

### Cotton miR397-LAC4 Module Participated in Defence-Induced Lignin Biosynthesis

**FIGURE 7:**
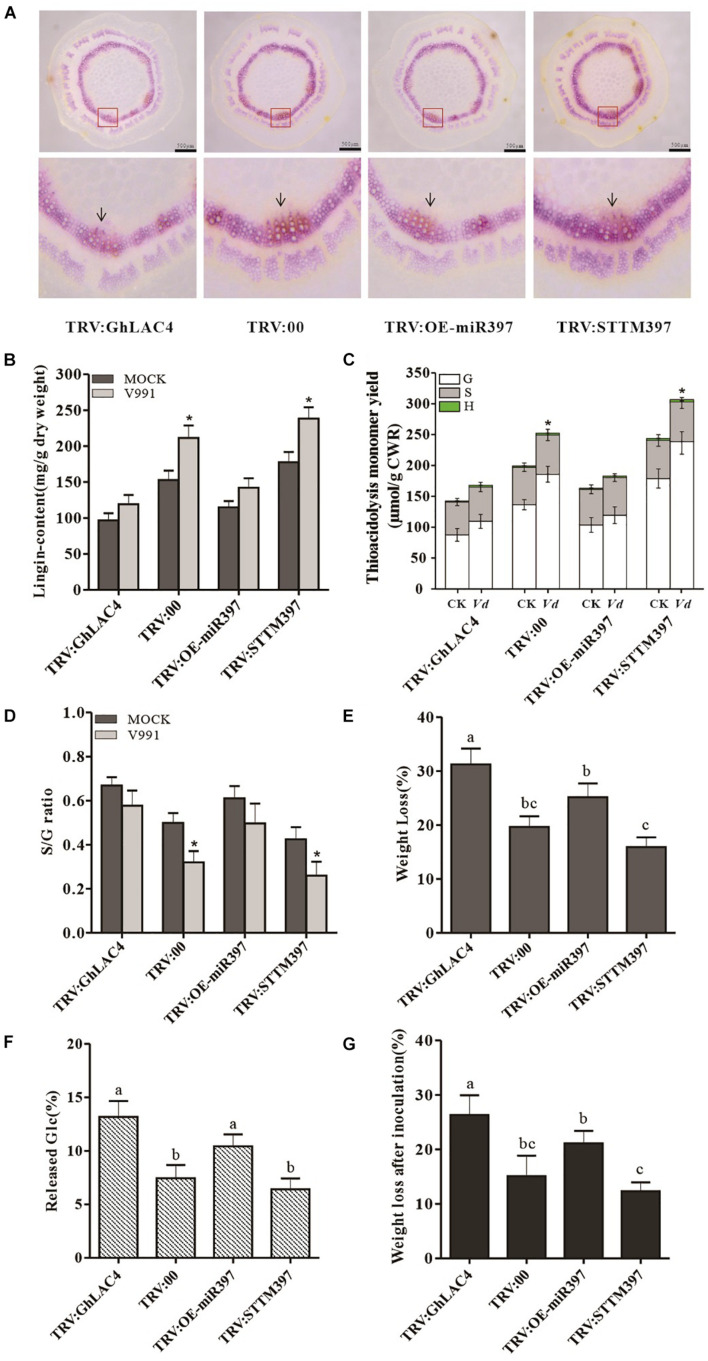
Analysis of defence-induced lignin under *V. dahliae* infection and lignin protection to cell wall degradation. **(A)** Phloroglucinol-HCL staining of stem cross slices in TRV:STTM397, TRV:OE-miR397, TRV:GhLAC4, and the control plants inoculated with *V. dahliae*. **(B)** The Klason lignin content of in TRV:STTM397, TRV:OE-miR397, TRV:GhLAC4, and the control plants inoculated with *V. dahliae*. **(C)** Determination of the main H, G, and S thioacidolysis monomers released by the lignins of extract-free mature stems of laccase in TRV:STTM397, TRV:OE-miR397, TRV:GhLAC4, and the control plants. **(D)** S/G molar ratio in in TRV:STTM397, TRV:OE-miR397, TRV:GhLAC4, and the control plants. **(E)** Weight loss of extract-free stems digested by cellulose in TRV:STTM397, TRV:OE-miR397, TRV:GhLAC4, and the control plants. **(F)** Glucose (Glc) released in reaction solution of extract-free stems digested by cellulose in TRV:STTM397, TRV:OE-miR397, TRV:GhLAC4, and the control plants. **(G)** Weight loss extract-free stems digested by cellulase digested by *V. dahliae* secretion in TRV:STTM397, TRV:OE-miR397, TRV:GhLAC4, and the control plants. Scale bars in **(A)** is 500 μm. Mean with SD comes from triple repeats experiments. Significant differences in **(B–D)** were determined using Student’s *t*-test (*P < 0.05). Different letters in **(E–G)** indicate significant difference (*P* < 0.05) based on Tukey’s HSD test.

Given that *GhLAC4* expression was induced in plants inoculated with *V. dahliae*, we hypothesised that lignin biosynthesis could be induced to enhance plant resistance. To prove this possibility, the induced lignin was first tested in VIGS plants inoculated with *V. dahliae* through phloroglucinol HCl staining. As shown in Figure 7A, as similar as the results in normal plants (Figure 6A), the red coloured staining indicating xylem in TRV:GhLAC4 and TRV:OE-miR397 plants was lighter compared to the control, while that in TRV:STTM397 showed a darker red colour. However, Klason lignin content was significantly higher in TRV:STTM397 plants inoculated with *V. dahliae* compared to those inoculated with mock, while lignin contents in TRV:GhLAC4 and TRV:OE-miR397 plants under *V. dahliae* treatment were slightly higher than those plants treated with mock, indicating that *GhLAC4* plays an important role in defence-inducible lignin biosynthesis (Figure 7B). According to GC/MS analysis G-lignin contents in TRV:STTM397 plants under pathogen treatment was significantly higher than those in the mock control, whereas S-lignin and H-lignin in these plants treated with pathogen showed comparable contents with those under mock treatment (Figure 7C). And G-lignin contents in TRV:GhLAC4 and TRV:OE-miR397 plants inoculated with pathogen were similar to these plants treated with mock, suggesting that *GhLAC4* acts in G-lignin biosynthese under *V. dahliae* induction as well as action in basal G-lignin biosynthesis. The S-lignin/G-lignin ratio in TRV:STTM397 plants inoculated with *V. dahliae* was lower than these plants under the mock treatment, while the S-lignin/G-lignin ratios in TRV:GhLAC4 and TRV:OE-miR397 plants inoculated with pathogen were comparable with these plants treated with mock (Figure 7D). These results demonstrated that *GhLAC4* participated in defence-induced lignin biosynthesis via the regulation of ghr-miR397, increasing plant resistance to *V. dahliae*.

### Plant Lignin Protected Cell Wall From Degradation by Hydrolases

Some studies have documented that plant lignin is a defensive physical/chemical barrier that prevents pathogenic invasion (Bonello and Blodgett, 2003; Zhang et al., 2017). Therefore, to determine how lignin protects the cell wall from degradation by hydrolases secreted from pathogens, commercial cellulase, a mimic of pathogen hydrolase, was employed to analyse cell wall degradation. The extract-free stems of VIGS plants were digested in a cellulase buffer solution. The results showed that the weight loss of extract-free samples obtained from TRV:GhLAC4 and TRV:OE-miR397 plants was significantly higher than that observed for the control plants, exhibiting 31.22% and 25.15% vs. 19.63% weight loss, respectively. However, the extract-free stems in TRV:STTM397 showed significantly lower weight loss (15.91%) than that observed for the control stems (Figure 7E). To further verify the saccharification potential of various extract-free stems by cellulase, the amounts of glucose (Glc) in the digestion solutions were measured. The results were consistent with the weight loss of extract-free samples, that is, Glc content in solutions was positively correlated with the weight loss observed for the extract-free samples (Figure 7F). These data on weight loss in TRV:GhLAC4, TRV:OE-miR397, and TRV:STTM397 plants digested by cellulase were negatively correlated with their lignin contents, indicating that lignin protects the cell wall from degradation by hydrolases. To confirm the function of lignin in protecting the cell wall from hydrolases, a parallel experiment was performed. The *V. dahliae* solution cultured for 5 days was filtered to obtain a pathogen-free solution containing pathogen secretion, which was used to digest extract-free stems instead of cellulase. In line with the results observed for cellulase degradation, higher lignin content in TRV:STTM397 plants could significantly reduce weight loss of extract-free samples compared to the control, while lower lignin content in TRV:GhLAC4 and TRV:OE-miR397 plants significantly increased weight loss of samples (Figure 7G). The results demonstrated that lignin increased plant resistance against *V. dahliae* partially by protecting the cell wall from hydrolases in pathogen secretion.

## Discussion

Plant lignin is deposited in the cell wall through the polymerization of monolignols by laccases (Berthet et al., 2011). Only 4 out of 17 laccase genes have been reported to act in lignin biosynthesis in *Arabidopsis* through genetic experiments (Wang et al., 2014). In addition, lignin can act as a barrier to prevent pathogenic invasion and enhance plant resistance (Bellincampi et al., 2014). However, the functions of *LAC* family genes participating in lignin biosynthesis and plant resistance have not been explored in the genetic analysis and regulation of miRNAs. Here, we characterised the role of cotton *LAC4* that acts in lignin biosynthesis by regulating miR397 and participating in plant defence.

*GhLAC4* can get directedly cleaved by ghr-miR397 in the post-transcriptional process as revealed by the degradome sequencing, specific amplicon analysis, and GUS reporter analysis. Additionally, GhLAC4 expression profiles showed a trend contrary to ghr-miR397 accumulation in both plant various tissues and plant response against *V. dahliae* infection. These results verify that ghr-miR397 can regulate GhLAC4 expression through post-transcriptional processes. Similar reports on the miR397-directed cleavage of LACs have been documented in *A. thaliana*. For instance, LAC4, a laccase gene, is regulated by miR397b, which controls lignin biosynthesis and seed yield in *A. thaliana*. Overexpression of miR397b (oxmiR397b) was found to reduce lignin deposition in transgenic plants (Wang et al., 2014). In *P. trichocarpa*, miR397a is known to be a negative regulator of laccase genes that affect lignin content (Lu et al., 2013). In rice, miR397-mediated laccase gene silencing can change lignification, facilitating plant erect growth and domestication of cultivated indica rice (Zhang et al., 2013; Swetha et al., 2018). Therefore, *LAC4* is also regulated by miR397 to participate in lignin biosynthesis in plants.

In this study, *GhLAC4* was found to act in lignin biosynthesis. *GhLAC4* knockdown plants showed lower lignin content and decreased G-lignin biosynthesis according to lignin content measurement, chemical staining, and GC/MS analysis. Although there are many LACs in plants, only a few of them participate in monolignol polymerisation into lignin (Niu et al., 2021). For example, of 17 laccase genes in *Arabidopsis*, only *LAC4*, *LAC11*, *LAC15*, and *LAC17* were verified to act in lignin biosynthesis according to genetic analysis (Liang et al., 2006; Berthet et al., 2011; Turlapati et al., 2011; Zhao et al., 2013). Recently, cotton *LAC15* was reported to participate in lignin biosynthesis and change the ratio of G-lignin/S-lignin (Zhang et al., 2019). Thus, *GhLAC4* is another laccase that directly acts in cotton lignin biosynthesis, which is consistent with the functional patterns of *LAC4* and *LAC15* in *Arabidopsis* (Wang et al., 2014; Zhang et al., 2019).

**FIGURE 8:**
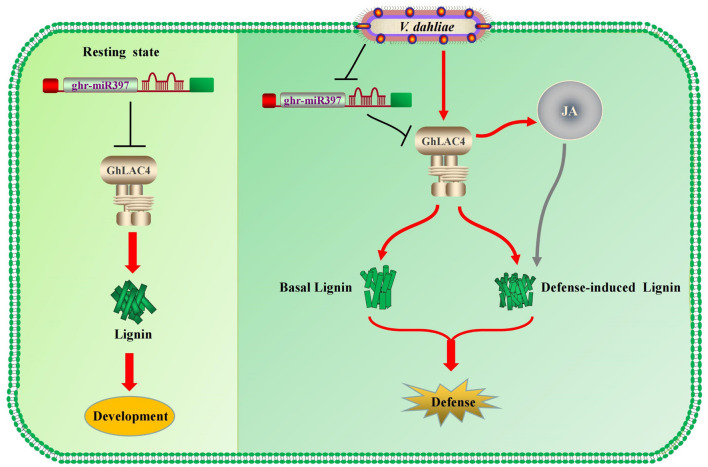
The cotton miR397-LAC4 module working pattern diagram. In resting state, *GhLAC4* normally acts in lignin biosynthesis **(Left)**. As infected by *V. dahliae*, the ghr-miR397-GhLAC4 module can mediate lignin accumulation through basal lignin biosynthesis and defence-induced lignin biosynthesis to increase plant defence **(Right)**.

Under the invasion of *V. dahliae*, *GhLAC4* expression levels increased, while ghr-miR397 accumulation decreased, leading to enhanced plant resistance. Lignin content significantly increased in VIGS plants infected with *V. dahliae* compared to the mock treatment (Figures 6D, 7B), indicating the biosynthesis of defence-induced lignin. These defence-induced lignin biosynthesis changes in VIGS plants were associated with JA signalling pathway. Increasing evidence has shown that some plant LACs, including those involved in basal lignin biosynthesis, respond to pathogenic invasion to increase cell wall lignification (defence-induced lignin), leading to an increase in plant defence (Nenaah, 2014; Mechri et al., 2015; Wang et al., 2015). For instance, overexpression of *GhLAC1* can enhance the lignification of cotton and increase its tolerance to biological stress (Hu et al., 2018). Overexpression of *GhLAC15* can increase the lignification and lignin content of plant cell walls, thus enhancing the resistance of plants against *V. dahliae* (Zhang et al., 2019). Therefore, in resting state, *GhLAC4* normally acts in lignin biosynthesis, which is deposited in the cell wall for plant growth and development; as infected by *V. dahliae*, the ghr-miR397-GhLAC4 module can mediate lignin accumulation through basal lignin biosynthesis and defence-induced lignin biosynthesis to increase plant defence, as shown in Figure 8.

In this study, we characterise a novel cotton LAC protein, *GhLAC4* in plant lignin biosynthesis and defence. *GhLAC4* is directedly cleaved by ghr-miR397 through post transcriptional processing. Ghr-miR397-GhLAC4 respond to *V. dahliae* infection and participate in plant defence. *GhLAC4* can act in basal lignin biosynthesis in resting state, and promotes inducible lignin biosynthesis when plant is invaded by *V. dahliae*. Both lignins can protect plant cell wall from degradation by hydrolases secreted by the fungi to increase plant defence.

## Data Availability Statement

The raw data supporting the conclusions of this article will be made available by the authors, without undue reservation, to any qualified researcher.

## Author Contributions

JW and YL conceived and designed the experiments. TW, YT, PJ, YQ, and AC performed the experiments. YZ, PW, and BW analysed the data and results. JW and TW wrote the manuscript. All authors read and approved the final manuscript.

## Conflict of Interest

YQ and AC were employed by the company Join Hope Seeds Co. Ltd. The remaining authors declare that the research was conducted in the absence of any commercial or financial relationships that could be construed as a potential conflict of interest.

## Publisher’s Note

All claims expressed in this article are solely those of the authors and do not necessarily represent those of their affiliated organizations, or those of the publisher, the editors and the reviewers. Any product that may be evaluated in this article, or claim that may be made by its manufacturer, is not guaranteed or endorsed by the publisher.

## Footnotes

1
https://www.cottongen.org/icgi/home

2
https://www.arabidopsis.org/
