# Supplementary material for: A Cotton Lignin Biosynthesis Gene, GhLAC4, Fine-Tuned by ghr-miR397 Modulates Plant Resistance Against Verticillium dahliae
Source: Front Plant Sci. 2021 Nov 18;12:743795. doi: 10.3389/fpls.2021.743795 (PMC8636836; doi:10.3389/fpls.2021.743795)
Supplement: Supplementary file 1 [file Data_Sheet_1.pdf]

Supplementary Figures and Table

A cotton lignin biosynthesis gene, *GhLAC4*, fine-tuned by ghr-miR397 modulates plant resistance against *Verticillium dahliae*

Taiping Wei[1,2], Ye Tang[2], Pei Jia[2], Yanming Zeng[2], BingTing Wang[2], Pan Wu[2], Yonggang Quan[3], Aimin Chen[3],Yucheng Li[1]*, Jiahe Wu[1,2]*

[1]School of Resources and Environmental Engineering, Anhui University, Anhui province, Hefei, 230601, China

[2]State Key Laboratory of Plant Genomic, Institute of Microbiology, Chinese Academy of Sciences, Beijing, 100101, China

[3]The Key Laboratory for the Creation of Cotton Varieties in the Northwest, Ministry of Agriculture, Join Hope Seeds CO. Ltd, Changji, Xinjiang, 831100, China

*** Corresponding Authors:**

Wu, Jiahe, wujiahe@im.ac.cn; Li, Yucheng, li-yucheng@163.com

Running title: *GhLAC4* biosynthesis lignin increasing defense

Supplementary Figure S1

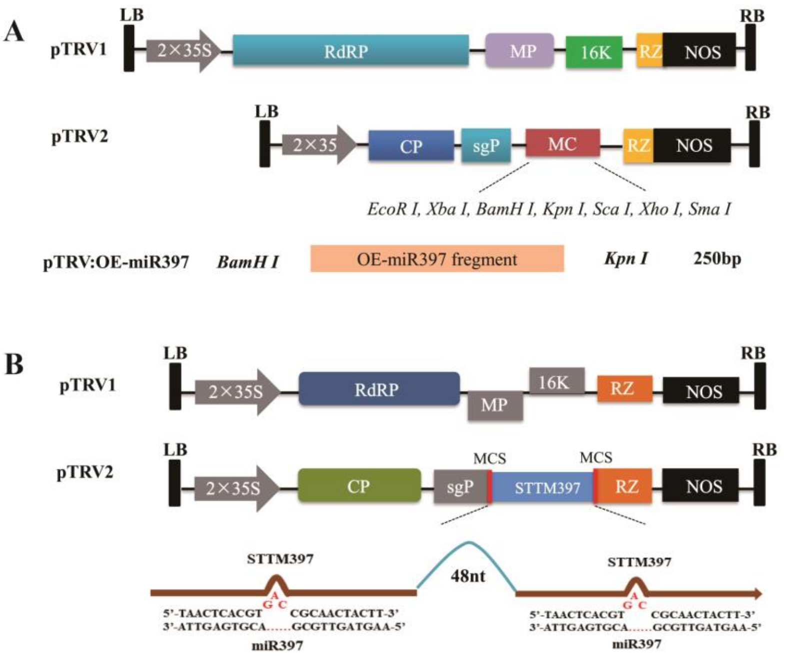

Supplementary Figure S1. Schematic diagram of ghr-miR397 overexpression and silencing vectors.

(A) Schematic diagram of ghr-miR397 overexpression vector. (B) Schematic diagram of ghr-miR397 silencing vector.

Supplementary Figure S2

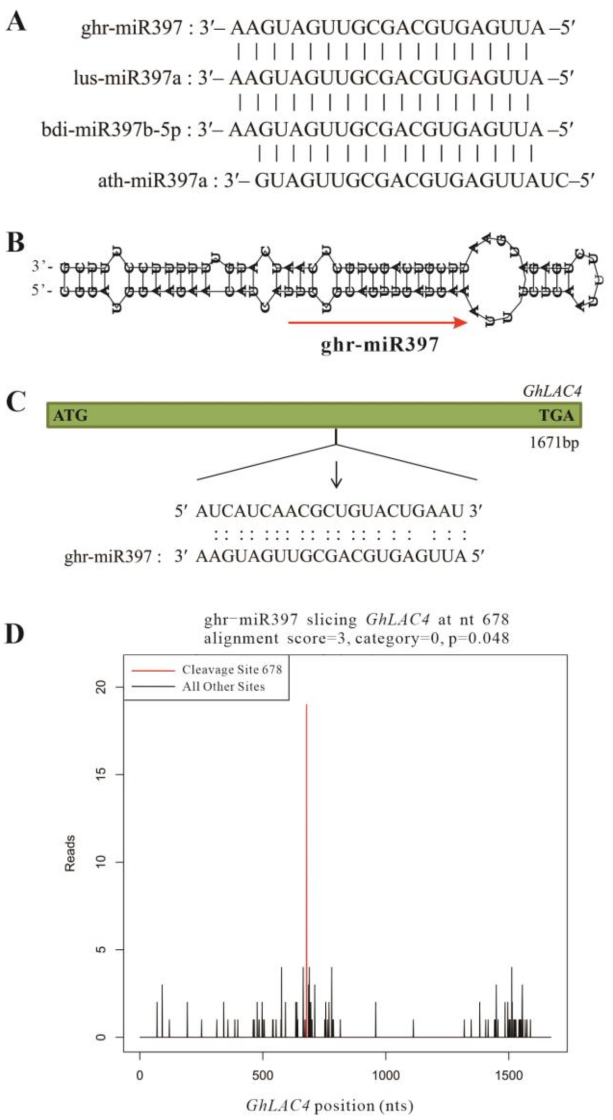

Supplementary Figure S2. Ghr-miR397 nucleotides and base complementary with *GhLAC4* and degradome analysis.

(A) The alignment of ghr-miR397 nucleotides with miR397 from *Linum usitatissimum*, *Brachypodium distachyon* and *Arabidopsis thaliana*. (B) Stem-loop structure of the ghr-miR397 precursor. The red arrow underlined indicated the mature sequence of miR397. (C) *GhLAC4* mRNA is complementary to ghr-miR397, and the black arrow indicates the cleavage site of the target gene. (D) T-shaped plots of ghr-miR397 and *GhLAC4* were verified by degradome sequencing. The T-plot shows the position of degradome markers in the full length of the target mRNA sequence.

Supplementary Figure S3

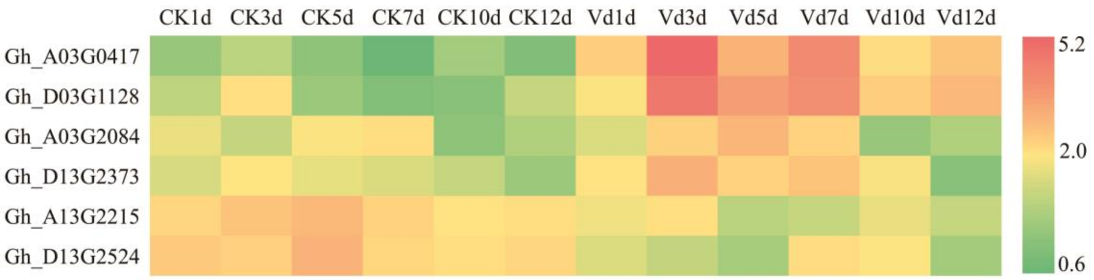

Supplementary Figure S3. The expression analysis of six targeted LAC genes under *V. dahliae* infection.

Heatmaps of differently expressed target genes in the 1, 3, 5, 7, 10 and 12 days postinoculation samples compared with the control. The color bar represents the relative signal intensity values from red (upregulated) to green (downregulated), indicating a range of 0.6 to 5.2.

Supplementary Figure S4

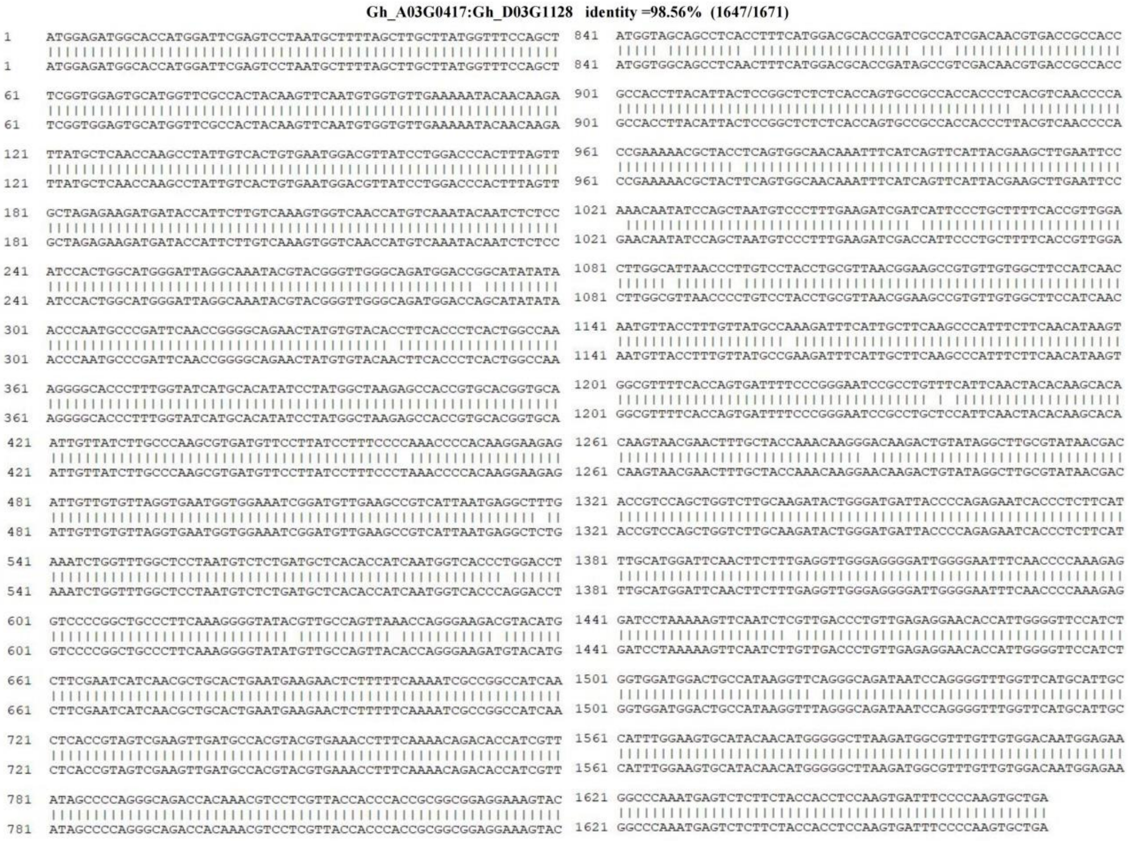

Supplementary Figure S4. Alignment of Gh_A03G0417 and Gh_D03G1128 nucleotide sequences.

Supplementary Figure S5

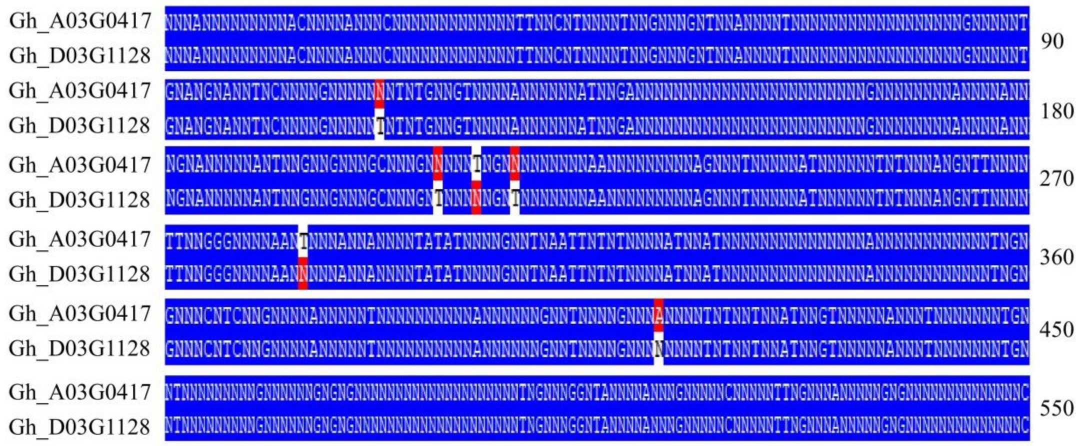

Supplementary Figure S5. Alignment of Gh_A03G0417 with Gh_D03G1128 amino acid sequence.

Supplementary Figure S6

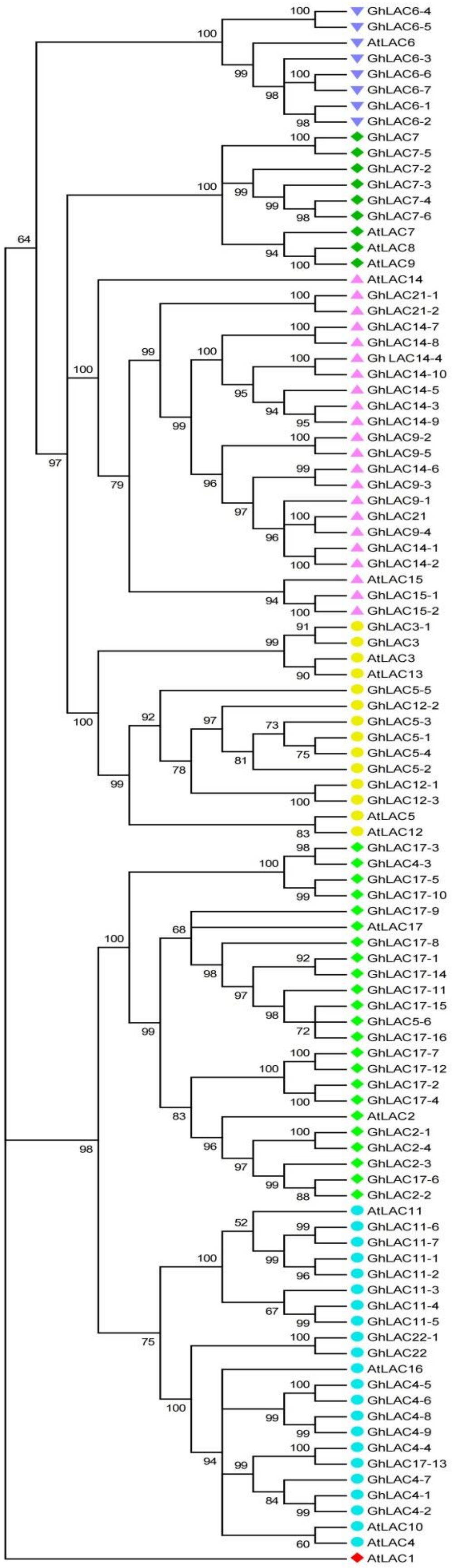

Supplementary Figure S6. Phylogenetic tree analysis of LACs gene family in *G. hirsutum* and *A. thaliana.*

It was performed using MEGA(Version7.1) software. According to different colors, they were divided into 6 groups, including fluorescent green group 1, sky blue group 2, yellow group 3, pink group 4, dark green group 5 and blue purple group 6, GhLAC4-9 and GhLAC4-8 are indicated in red box.

Supplementary Figure S7

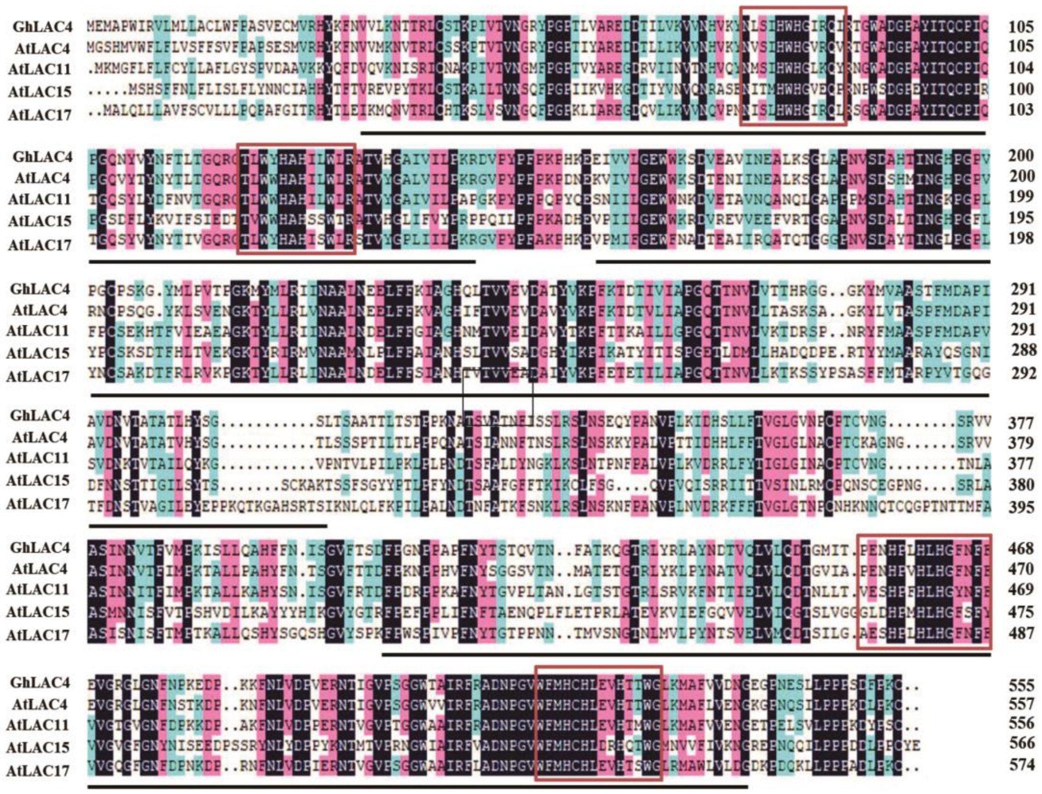

Supplementary Figure S7. Amino acid sequence alignment between GhLAC4 and four *Arabidopsis* laccases acting in lignin biosynthesis.

The black lines represent the oxidase domains and the red boxes represent the copper ion binding domains of laccases.

Supplementary Figure S8

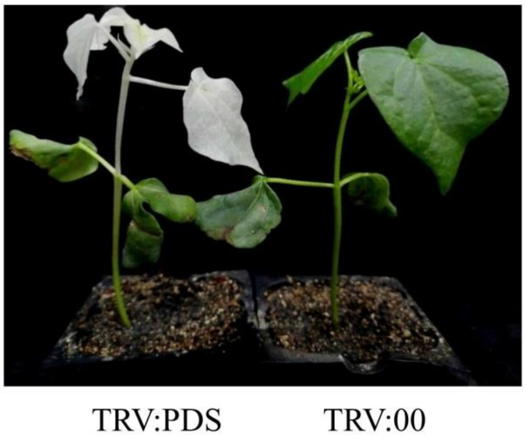

Supplementary Figure S8. Photo-bleaching leaves of *GhPDS*-silenced plants.

Supplementary Figure S9

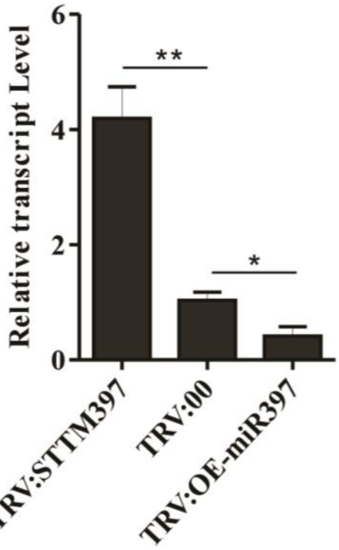

Supplementary Figure S9. Relative expression levels of *GhLAC4* in TRV:STTM397, TRV:OE-miR397 and TRV:00 plants. Mean with SD comes from triple repeats experiments. Significant differences were determined using Student's *t*-test (*$P < 0.05$, **$P < 0.01$).

Supplementary Figure S10

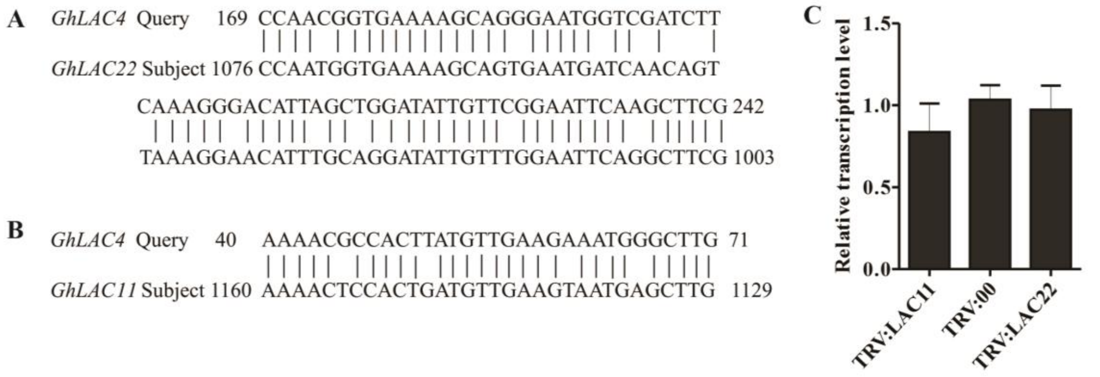

Supplementary Figure S10. The *GhLAC11* and *GhLAC22* expression levels in GhLAC4-silenced plants.

(A) The alignment of *GhLAC4* fragment nucleotides with *GhLAC22*. (B) The alignment of *GhLAC4* fragment nucleotides with *GhLAC11*. (C) Relative expression levels of *GhLAC11* and *GhLAC4* in the GhLAC4-silenced plants compared to the TRV:00 plants.

Supplementary Table S1. Primers used in the present research

	A	B	C
1	Primer name	Sequence(5′-3′)	destination
2	LAC4-F	AGAAGGCCTCCATGGGGATCCTTGAATGGAGCAGGCGGA	VIGS
3	LAC4-R	GAGACGCGTGAGCTCGGTACCCGTCAACCCCACCGAAAAA	VIGS
4	OE-miR397-F	AGAAGGCCTCCATGGGGATCCATCCCCGGATGGAAGAAACA	overexpression
5	OE-miR397-R	GAGACGCGTGAGCTCGGTACCAATTTACTTTTCAATTGTTCCAAAGG	overexpression
6	STTM397-F	AACGAGTCTAGAGGATCCTAACTCACGTGACCGCAACTACTTGTTGTTGTTGTTATGGTC	STTM
7	STTM397-R	CGGGCCTCGAGGGTACCAAGTAGTTGCGGTCACGTGAGTTAATTCTTCTTCTTTAGACCA	STTM
8	V-PDS-F	GAAGGAGCTGAATATCACTCCC	VIGS
9	V-PDS-R	ATTAACACCGTTGCGGCTAAGC	VIGS
10	qLAC4-F	GACCATTCCCTGCTTTTCACC	qRT-PCR
11	qLAC4-R	TGCTTGTGTAGTTGAATGGAGC	qRT-PCR
12	qmiR397-F	CGCGATTGAGTGCAGCGTT	qRT-PCR
13	qmiR397-R	AGTGCAGGGTCCGAGGTATT	qRT-PCR
14	qLAC22-F	GAGACGCGTGAGCTCGGTACCAAGGCATCAAAGGTTTTCCAGG	qRT-PCR
15	qLAC22-R	AGAAGGCCTCCATGGGGATCCGCCGAATCTCTTCGAAGCCT	qRT-PCR
16	qLAC11-F	GAGACGCGTGAGCTCGGTACCTACCAAGATTTGCAGTCAATGGTG	qRT-PCR
17	qLAC11-R	AGAAGGCCTCCATGGGGATCCGCAAATGTGCCTCTTAAAGTTGAC	qRT-PCR
18	qU6-F	CGGGGACATCCGATAAAATTGGAAC	qRT-PCR
19	qU6-R	GGACCATTTCTCGATTTGTGCGTG	qRT-PCR
20	V.dtubulin-F	AACAACAGTCCGATGGATAATTC	qRT-PCR
21	V.dtubulin-R	GTACCGGGCTCGAGATCG	qRT-PCR
22	qUB7-F	GAAGGCATTCCACCTGACCAAC	qRT-PCR
23	qUB7-R	CTTGACCTTCTTCTTCTTGTGCTTG	qRT-PCR
24	RemiR397	GTCGTATCCAGTGCAGGGTCCGAGGTATTCGCACTGGATACGACTTCATC	Reverse transcription
25	qPR1-F	AAGGCCGACTACGATTACAGC	qRT-PCR
26	qPR1-R	CGCCATTGTTGCACTTCACC	qRT-PCR
27	qPR3-F	GATGACTCCACAATCACCGAAGC	qRT-PCR
28	qPR3-R	GCGGTCTTCTACCTGGGCATT	qRT-PCR
29	qPR5-F	TGGTCCGACCCGTTATTCAA	qRT-PCR
30	qPR5-R	CCCGAGGCTCACCTCAT	qRT-PCR

31	qPDF1.2-F	CTGTCCTAGCGGATGGTGATAAG	qRT-PCR
32	qPDF1.2-R	GTGCAGACGCATTTGCGAAGGAA	qRT-PCR
33	q4CL-F	AAGTGACGATTGCTCCGATTGT	qRT-PCR
34	q4CL-R	ACTGGTCCTGCCTCGGTCATAC	qRT-PCR
35	qCCoAOMT-F	TGGTGAAGGTTGGTGGTTTGAT	qRT-PCR
36	qCCoAOMT-R	CATGCAAATCTCAATCCTGGGG	qRT-PCR
37	qCOMT-F	ACGATTGGAGCGATGAGCACTG	qRT-PCR
38	qCOMT-R	AACCTTGAAACCCAGCACTCCT	qRT-PCR
39	qF5H-F	TGGATGTGATGTTCGGTGGGAC	qRT-PCR
40	qF5H-R	GATACCCCGCCACTATTGCCTC	qRT-PCR
41	qCAD-F	ACCCCTCTTCAGTTTGTTTCCC	qRT-PCR
42	qCAD-R	CATCGTTCTTCTCCAGCCTCTC	qRT-PCR
43	qC4H-F	AGAGGAAGAAGCTTGCGAGCAC	qRT-PCR
44	qC4H-R	GTTCACAAGCTCAGCAATGCCC	qRT-PCR
45	qHCT-F	CTGAAAGCACAGCAATCTCCAT	qRT-PCR
46	qHCT-R	CCAAAGTAACCAGGTGGGAGTG	qRT-PCR
47	qC3H-F	AAACGTGTGGCCGCGGATATTA	qRT-PCR
48	qC3H-R	ATCACTTGCTTATCGCGCCCTT	qRT-PCR
49	qC4H-F	AGAGGAAGAAGCTTGCGAGCAC	qRT-PCR
51	qC4H-R	GTTCACAAGCTCAGCAATGCCC	qRT-PCR
52	qCCR-R	AGGTGGAAAAAGTGGCCAAAGC	qRT-PCR
53	qCCR-R	GCCTCTTGCTGTCTTCGCACTA	qRT-PCR
54	LAC4-GUS-F	GAGAACACGGGGGACTCTAGAATGGAGATGGCACCA TGGATTC	transient expression
55	LAC4-GUS-R	ATAAGGGACTGACCACCCGGGGATCCGCACTTGGGG AAATCACTTGGA	transient expression
56	Pre-miR397-F	CCTGTCGACGGATCCTCTAGAATCCCCGGATGGAAG AAACA	transient expression
57	Pre-miR397-R	GGATACATGTACGTAACGCGTAATTTACTTTTCAATTG TTCCAAAGG	transient expression
58	mLAC4-GUS-F	TGCTTCGAATCATCAATGCAGCTTTGAATGAAGAACT CTT	transient expression
59	mLAC4-GUS-R	GTTCTTCATTCAAAGCTGCATTGATGATTCGAAGCAT GTAC	transient expression
60	LAC4F1	TGCCAGTTACACCAGGGAAG	transient expression
61	LAC4R1	ACGAGGACGTTTGTGGTCTG	transient expression
62	LAC4F2	GAAGAACTCTTTTTCAAAATCGCCG	transient expression
